# Complex and Unusual Excited-State Relaxation Dynamics of 9,9′-Bifluorenylidene Revealed by Comprehensive Time-Resolved Spectroscopy and MRSF-TDDFT Calculations

**DOI:** 10.1021/acs.jpclett.6c00465

**Published:** 2026-03-24

**Authors:** Chen Wang, Woojin Park, Cheol Ho Choi, Seogjoo J. Jang, Sri Harsha Mamillapalli, Jinjia Xu

**Affiliations:** ∇ Department of Chemistry and Biochemistry, Queens College, 14772 City University of New York, Queens, New York 11367, United States; ‡ The Graduate Center, 14772 City University of New York, New York, New York 10016, United States; § Department of Chemistry, 34986 Kyungpook National University, Daegu 41566, South Korea; ∥ Department of Chemistry and Biochemistry, University of Missouri−St. Louis, St. Louis, Missouri 63121, United States

## Abstract

Design of novel photochemical molecular motors often requires molecular building blocks that exhibit rather unusual photoactivity, for which conventional analyses of spectroscopic data can lead to conflicting interpretations. We here systematically investigated the excited-state relaxation dynamics of one such molecule, 9,9′-bifluorenylidene (**BF**), through comprehensive and complementary integration of ultrafast transient absorption (TA) and femtosecond stimulated Raman (FSRS) spectroscopies and first-principles mixed-reference spin-flip time-dependent density functional theory (MRSF-TDDFT). TA and FSRS identified two sequentially formed transients following photoexcitation. The decay kinetics of the two intermediates differ in response to excitation wavelengths and the viscosity/polarity of solvents. MRSF-TDDFT calculations reveal a direct, barrierless internal-conversion pathway from the bright Franck–Condon state to a dark S_1_ minimum, where the excited-state population is transiently trapped, accounting for the first transient species observed in spectroscopic experiments. Further tracking down along the PES with MRSF-TDDFT mapped out two nonradiative relaxation pathways via conical intersections that connect the dark S_1_ state to three configurations in the ground-state manifolds, within which a ring structure with a C8–C8′ bond and the vibrationally excited ground-state **BF** were identified from spectroscopic and kinetic data. The complexity of relaxation kinetics was attributed to the flexible torsional and twisting motions about the C9–C9′ bridge bond enabled by the diradical character of the S_1_ state. These findings clarify unusual photoactive relaxation dynamics stemming from a novel correlation between structural flexibility and shifting electronic characteristics, and they demonstrate the importance of integrating spectroscopic and advanced electronic structure calculation studies for judicious clarification of complex, competing relaxation pathways of excited states.

## Graphical abstract

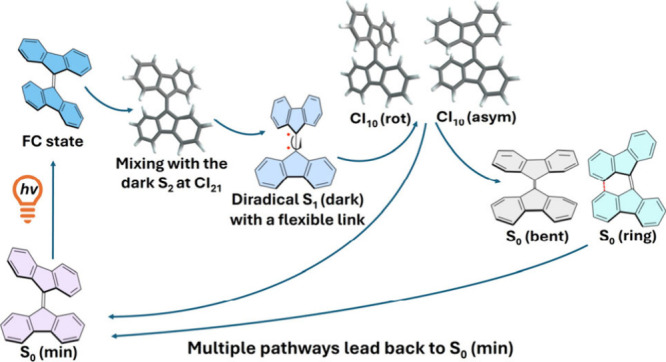

Light-responsive molecules can be used to fabricate novel molecular electronics and optoelectronics, such as organic field-effect transistors, molecular logic gates, high-density optical memory devices, and molecular wires.^1–4^ To improve molecules’ response to photo stimuli, it is essential to understand the fundamental mechanisms that regulate their energy relaxation, charge transfer, and emission. Photochemical molecular motors based on mechanically active multicyclic structures are particularly intriguing because they can undergo conformational and electronic changes in response to excitation and redox stimuli. Their dynamic behavior enables applications across various fields, including molecular machines, dynamic materials, and energy conversion systems.^5–8^ In particular, building blocks consisting of π-conjugated frameworks with rigid backbones can be interlocked via various chemical linkers to construct responsive chains with tunable photophysical properties for innovative functional applications, such as molecular actuators and soft robotics.^9,10^ However, unusual and complex excited-state dynamics exhibited by many of these molecules often make it challenging to make a clear interpretation of spectroscopic data.

As an overcrowded alkene system, 9,9′-bifluorenylidene (**BF**) exhibits a highly dynamic twisted status around its central C=C bonds due to pronounced steric congestion between the two fluorene moieties (Figure 1A). This twisting distortion reduces the π-overlap across the double bond, endowing **BF** with substantial diradical character that fluctuates with the torsional angle between the fluorene π-planes. Such a delicate balance between closed-shell and open-shell (diradical) configurations profoundly influences the molecule’s spin multiplicity, optical gap, and charge-transport characteristics.^11^ These properties make **BF** a promising building block for photovoltaic systems, singlet fission materials, and responsive molecular devices.^12–14^ Studies have shown that the structural and electronic flexibility of **BF** contributes to its nontrivial redox behavior and efficient energy transfer properties, which are critical for developing advanced optoelectronic systems.^15,16^ The torsional structural fluctuation in the excited-state manifold shows promise for applications of **BF** as a prototypical component in the construction of molecular machines that convert rotational motion into stretching,^17,18^ which can serve as a novel mechanism to direct molecular motions.

**Figure 1:**
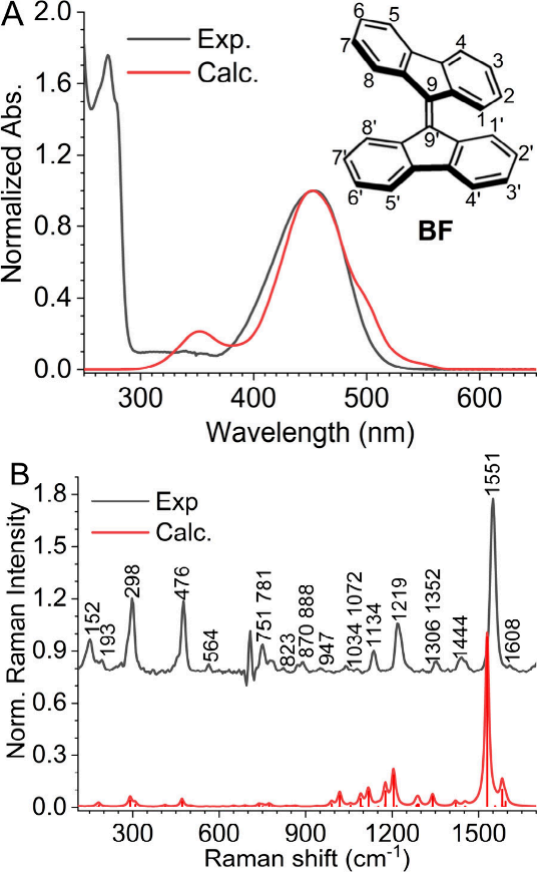
(A) Comparison of the experimental UV–vis spectrum of BF in *n*-hexane and the calculated absorption spectrum using MRSF-TDDFT by sampling the Wigner distribution (see Computational Methods section of the Supporting Information for details). The experimental and calculated spectra were normalized at their first absorption peak. A uniform 50 nm bathochromic shift was applied to match the calculated spectrum with the experimental absorption maxima. (B) Stimulated Raman spectrum of BF in hexane collected with a 522 nm Raman pump. Details of the stimulated Raman setup are provided in the Supporting Information (Figure S1, S2, and S3). The calculated Raman spectrum using DFT at the level of B3LYP/6-311++(d,p). The scaling factor for calculated frequencies is 0.9679.^24^ The calculated spectra were plotted with a line width of 4 cm^–1^.

Although there have been spectroscopic investigations of BF,^19^ key details of the excited-state relaxation dynamics of BF have not yet been clarified. In particular, a unified mechanistic picture that links transient spectral features to specific excited-state structures and relaxation pathways remains lacking, with significant implications for the use of these molecules as photoactive molecular motors. This motivates a more comprehensive and detailed spectroscopic and theoretical investigation of BF. In this work, we conduct such an investigation and uncover new and important details of the puzzling relaxation dynamics of the electronic excited state of **BF**. To be more specific, we here combine comprehensive kinetic data from time-resolved spectroscopies and detailed information from advanced quantum-mechanical calculations based on a recently developed mixed-reference spin-flip time-dependent density functional theory (MRSF-TDDFT).^20^ MRSF-TDDFT provides a balanced description of both open- and closed-shell configurations, such as diradicals, while correctly capturing the topology of conical intersections, all at a computational cost comparable to standard density functional theory. Femtosecond transient absorption (fs-TA) spectroscopy revealed multicomponent relaxation kinetics that point to indirect relaxation pathways involving an intermediate state, while femtosecond stimulated Raman spectroscopy (FSRS) captured vibrational signatures that belong to evolving transient intermediates during a sequential energy dissipation process. A systematic investigation of the dependence of relaxation kinetics on excitation energy and the solvation environment reveals detailed characteristics of the underlying relaxation pathways. MRSF-TDDFT calculations accurately reproduced the key experimental features of the excited-state and its relaxation pathways, with reasonable quantitative agreement. Beyond its fundamental relevance, **BF** serves as a model system for developing next-generation materials for dynamic photonic devices, advanced molecular sensors, and energy conversion technologies.^21–23^ By systematically exploring the effects of solvent viscosity, polarity, and excitation wavelength on the dynamic behavior of **BF** in its excited state, this work, for the first time, clarifies competing pathways in its relaxation process, between twisting and diradical-mediated ring-formation. The existence of the latter energy-dissipating pathway means that additional structural modifications of **BF** can be actively utilized for its application as molecular motors. These results also underscore the importance of detailed control over the excited-state properties of molecular systems for designing effective photoresponsive materials.

Figure 1 shows experimental and theoretical UV–vis and stimulated Raman spectra of **BF**. The steady-state absorption of **BF** in *n*-hexane exhibits a single absorption band in the visible centered at 454 nm (Figure 1A), which is reasonably modeled with our theoretical calculation. According to our MRSF-TDDFT calculations, this band corresponds to the vertical transition from the ground state to the Franck–Condon (FC) S_1_ state, which is dominated by the HOMO → LUMO excitation (3.136 eV). In addition, two nearly degenerate excited states, S_2_ (3.152 eV) and S_3_ (3.159 eV), also exist, arising mainly from the mixture of HOMO–2/HOMO–1 → LUMO transitions. Oscillator strengths of these transitions are listed in Table S1, and the relevant molecular orbitals are illustrated in Figure S4. Similar energetic features were obtained with different basis sets and exchange–correlation functionals. BH&HLYP was adopted for calculations because it is suitable for spin-flip methods that require a higher fraction of exact exchange. It was argued that rapid relaxation from the bright S_3_ state to the lower optical forbidden states through internal conversion was the reason for the low fluorescence quantum yield of **BF**.^19^ We will revisit the excited-state dynamics of **BF** in the following section. We acquired the ground-state Raman spectrum of **BF** with a 522 nm Raman pump, which is preresonant with the lowest optically allowed transition of **BF** (Figure 1B). Assignments of normal vibrational modes for the major Raman bands of **BF** are based on DFT (B3LYP/6-311++G­(d, p)) calculations and are listed in Table S2. **BF** showed sharp Raman peaks at 1551, 1219, 1134, 476, 298, 193, and 152 cm^–1^, which agree with the previously reported resonance Raman spectrum of this compound.^25^ We noticed that the strong low-frequency modes in **BF** spectra at 476 and 298 cm^–1^, corresponding to C–H wagging movements in or out of the fluorenylidene planes.

TA spectra and kinetics of **BF** were previously reported by Meech and Co-authors,^19^ but we observed the evolution of transient species over a broader spectral range, from 430 to 770 nm, revealing new aspects. The TA experiments were also carried out in different solvent systems to investigate the effects of solvent viscosity and polarity on the relaxation process of photoexcited **BF**. In addition, time-resolved Raman spectroscopy was conducted to probe different electronic states, and their relaxation pathways were mapped using MRSF-TDDFT calculations. Taken together, these provide a comprehensive and detailed interpretation of the excited-state dynamics of **BF**.

In *n*-hexane, upon 420 nm pump excitation, TA produced a signal of ground-state bleaching (GSB) that resembles the steady-state absorption profile, a broad excited-state absorption (ESA) band centered at around 683 nm, and an ESA plateau feature evolving between 550 and 500 nm (Figure 2A). Kinetic traces were extracted for these three representative spectral features and fitted with exponential decays, as shown in Figure 2B and listed in Table 1. Modeling of the GSB signal at 454 nm revealed a dual-stage recovery with lifetimes of 4.5 and 13 ps, suggesting an indirect relaxation process involving intermediate states. The two exponential components account for 64% and 36% of the GSB amplitude, respectively. On the ESA side, the 683 nm signal was fitted well by a single-exponential decay with a lifetime of 4.9 ps, comparable to the first decay time in the GSB signal. Following the decay of the 683 nm ESA, the 550 nm ESA plateau gradually declines and evolves to a new ESA band centered at 500 nm. We chose the kinetic trace at 503 nm to reflect the evolution of the new ESA band, and extracted a growth with a time constant of 4.0 ps followed by a decay with a lifetime of 15 ps.

**Figure 2:**
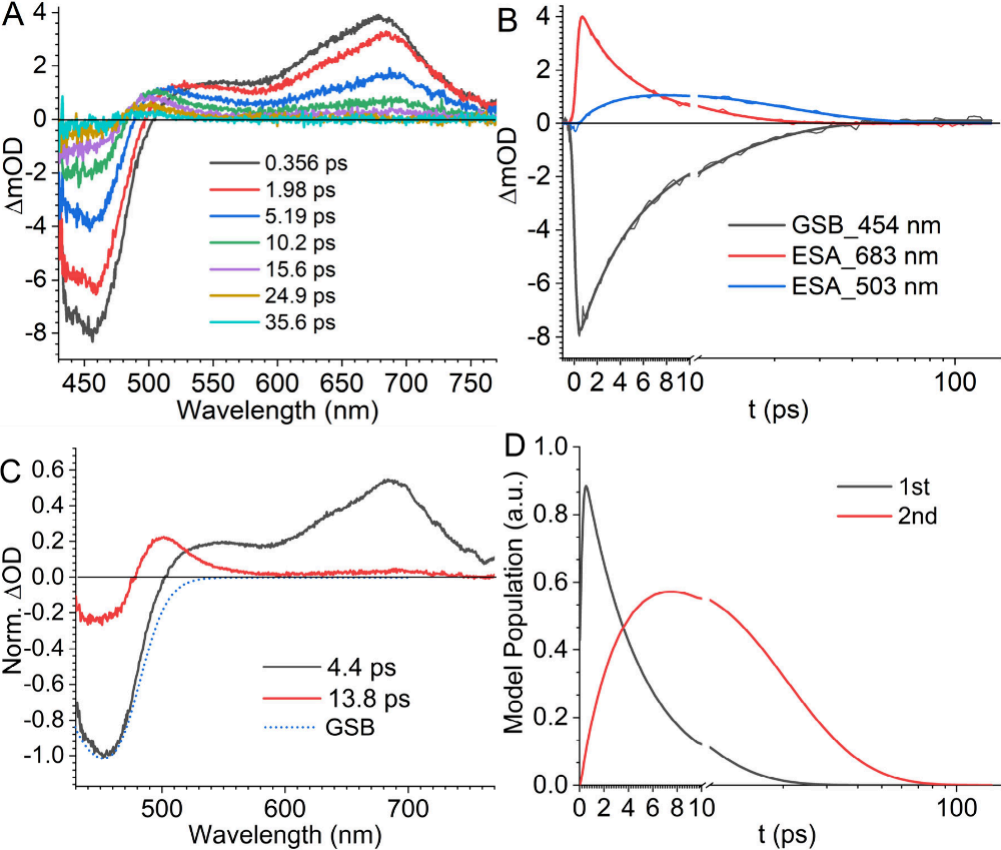
Excited state dynamics of **BF** in *n*-hexane recorded by fs-TA with a 420 nm pump. (A) fs-TA spectra at selective time delays. (B) TA kinetic traces extracted at 454, 683, and 503 nm. Exponential fitting parameters are listed in Table 1. (C) Species-associated spectra and (D) the kinetics of transient populations extracted from global analysis of the fs-TA data with a three-state, sequential decay model. The spectra were scaled by normalizing the GSB minimum to −1.0, and the species lifetimes were labeled in the legend. A reserved ground state absorption spectrum was scaled to match the minimum amplitude of the negative signal for comparison.

**Table 1:** Exponential Fitting Results for Kinetic Traces in Figure 2B^a^

**Signals (nm)**	**τ_1_ (ps),** **A_1_ (%)**	**τ_2_ (ps),** **A_2_%**
GSB 454	4.5 ± 0.8, –64 ± 11	13 ± 3, –36 ± 11
ESA 683	4.9 ± 0.1	
ESA 505	4.0 ± 0.5	15 ± 2
	Growth	Decay

a Errors were generated from the kinetic fitting.

To better understand the evolution of TA spectra, we applied global analysis to fit the TA data using a sequential-decay model involving two transient species (Figure 2C). The model showed that the initially observed species, with ESA signals at 683 and 550 nm, evolved into a second species characterized by an ESA signal at 500 nm with a time scale of 4.4 ps. These second species then decay over 13.8 ps. Global analysis also indicated that the second species has a weak ESA in the near-IR region (>700 nm). A decay model with parallel pathways was also tested to fit the data, but spectra generated by this model cannot capture features of specific intermediate states, as shown in Figure S5. A subtle dependence of relaxation kinetics on excitation energy was observed when the actinic pump wavelength was changed from 420 to 475 nm, as illustrated in Figure S6. Global analysis of the TA data reveals that the decay rate of the initial excited species slightly slowed from (4.4 ± 0.1 ps)^−1^ to (5.2 ± 0.1 ps)^−1^, while the decay of the second species remained unaffected (Table 2).

**Table 2:** Lifetimes of Two Relaxation Intermediates Obtained from Global Analysis for TA Collected in Different Solvents with Different Excitation Wavelengths

**Solvent**	**Viscosity**, **μ 25** ^**o**^ **C (cP)**	**Polarity** ^a^ π*	**λ**_**Ex**_ **(nm)**	**τ**_**1**_ **(ps)** ^b^	**τ**_**2**_ **(ps)** ^b^
*n*-Hexane	0.31	–0.04	420 nm	4.4 ± 0.1	13.8 ± 0.1
			475 nm	5.2 ± 0.1	13.8 ± 0.1
Cyclohexane	1.0	0	420 nm	5.1 ± 0.1	12.8 ± 0.1
			475 nm	6.0 ± 0.1	13.5 ± 0.1
CHCl_3_	0.56	0.58	420 nm	5.1 ± 0.1	18.8 ± 0.2
Acetonitrile	0.38	0.75	420 nm	4.6 ± 0.1	18.2 ± 0.2

a Kamlet and Taft’s scale, π*.

b Errors were estimated by global fitting.

We interrogated the dependences of **BF** relaxation kinetics on solvent properties. The excited-state behaviors of **BF** were compared for four solvents, *n*-hexane, cyclohexane, chloroform (CHCl_3_), and acetonitrile, which represent different viscosities and polarities, respectively. The trend of kinetic changes with the solvents is illustrated as single-wavelength traces in Figure S7. In all solvents, the relaxation process can be described as a sequential decay of two transient species, with a slight bathochromic shift and spectral broadening observed in the ESA of the initial excited species as solvent polarity increases. Kinetic parameters extracted from the global analysis using a three-state, sequential decay model (Figure S8) are summarized in Table 2. The relaxation rate of the first excited species slightly decreases with the increase in solvent viscosity. When the viscosity increases from 0.31 cP (*n*-hexane) to 1.0 cP (cyclohexane), the lifetime increases from 4.4 ± 0.1 ps to 5.1 ± 0.1 ps with 420 nm pump excitation. The 475 nm excitation experiment shows the same trend, with the lifetime increasing from 5.2 ± 0.1 ps in *n*-hexane to 6.0 ± 0.1 ps in cyclohexane (Figure S8). On the other hand, the lifetime of the second transient species is less sensitive to viscosity but prominently elongates with the increase of the solvent polarity. Compared to nonpolar *n*-hexane and cyclohexane, the lifetimes of the second species in CHCl_3_ and acetonitrile increase from about 13 ps to >18 ps. The dependence of lifetimes of the first and second relaxation intermediates on different conditions is illustrated in Scheme 1.

**Scheme 1:**
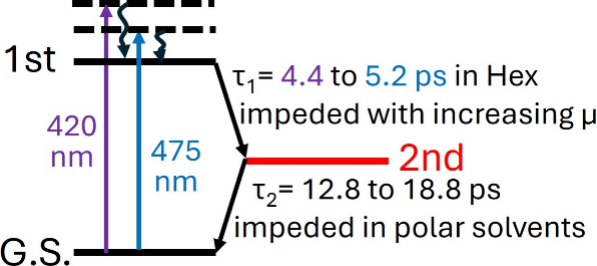
Trend of Kinetic Parameters Changes with Experimental Conditions

Femtosecond stimulated Raman spectroscopy (FSRS) confirmed the existence of the two transient species of **BF** observed in the TA experiment (Figure 3). The 522 nm Raman pump used in the FSRS experiment is on resonance with the ∼550 nm transition of the initial species and the ∼500 nm transition of the second transient species. The initial species is prominent in the spectrum acquired at a 1 ps time delay after the actinic pump, which shows Raman signals at 1510, 1445, 1301, 1044, 816, and 177 cm^–1^. The 1445 cm^–1^ band can be regarded as the characteristic signal of the initial state, owing to its relatively strong intensity and its lack of overlap with strong ground-state signals. The 1445 cm^–1^ band attenuates and disappears within 10 ps. In the meantime, another group of Raman signals, including the 1539, 1208, 468, 290, and 148 cm^–1^ bands, continues to grow and reaches a maximum at 5 ps. We note that these Raman signals resemble bathochromically shifted vibrational bands in the ground-state Raman spectrum shown in Figure 1B, which strongly suggest that they originate from species in the ground electronic state. In spectra collected after 5 ps time delay, a few new signals are also distinguished at 1075, 1001, 947, and 863 cm^–1^, as highlighted in blue in Figure 3. Based on their appearance times, we assign the two groups of bands to the second transient species observed in TA, which exhibits an ESA of ∼500 nm.

**Figure 3:**
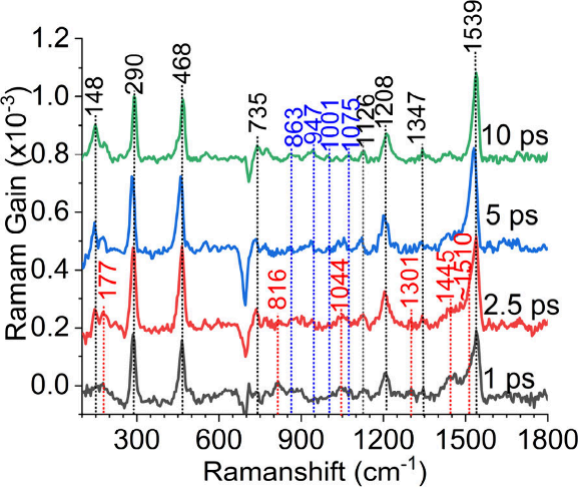
FSRS spectra of **BF** in n-hexane collected at different time delays after the actinic pump pulse. The red dotted lines denote signals assigned to the first excited states, and the black and blue dotted lines denote signals belonging to the second transient species.

We employed MRSF-TDDFT to elucidate the relaxation pathway of **BF** in the excited-state manifold by mapping the relevant potential energy surfaces (PESs). As shown in Figure 4A, the calculations indicate that the initially photoexcited bright S_1_ state in the Franck–Condon (FC) region undergoes barrierless relaxation to a nearby local minimum on the same S_1_ (bright) surface, and subsequently toward a conical intersection (CI_21_), where strong electronic mixing with an upper-lying dark S_2_ state occurs. This interaction yields a new S_1_ state characterized by an almost vanishing oscillator strength (f = 0.0003) to the S_0_ state. Such a state-mixing process and shallow energy barrier from S_1_ (bright) to CI_21_ effectively suppress direct radiative decay from the S_1_ (bright) state and redirects the excited-state population toward the nonradiative global minimum region on the S_1_ PES (S_1_ (dark)). The dark character of this global minimum arises from the spatial separation of the dominant HOMO–1 → LUMO transition, in which the HOMO–1 orbital is localized on one fluorenylidene unit and the LUMO is centered at the C9–C9′ bridge, leading to a negligible orbital overlap. The rapid establishment of this dominant S_1_ (dark) population in the early stages of excited-state relaxation is fully consistent with the experimentally observed extremely low fluorescence quantum yield of the compound.^19^ We assign the first transient intermediates observed by TA to the S_1_ (dark) state.

**Figure 4:**
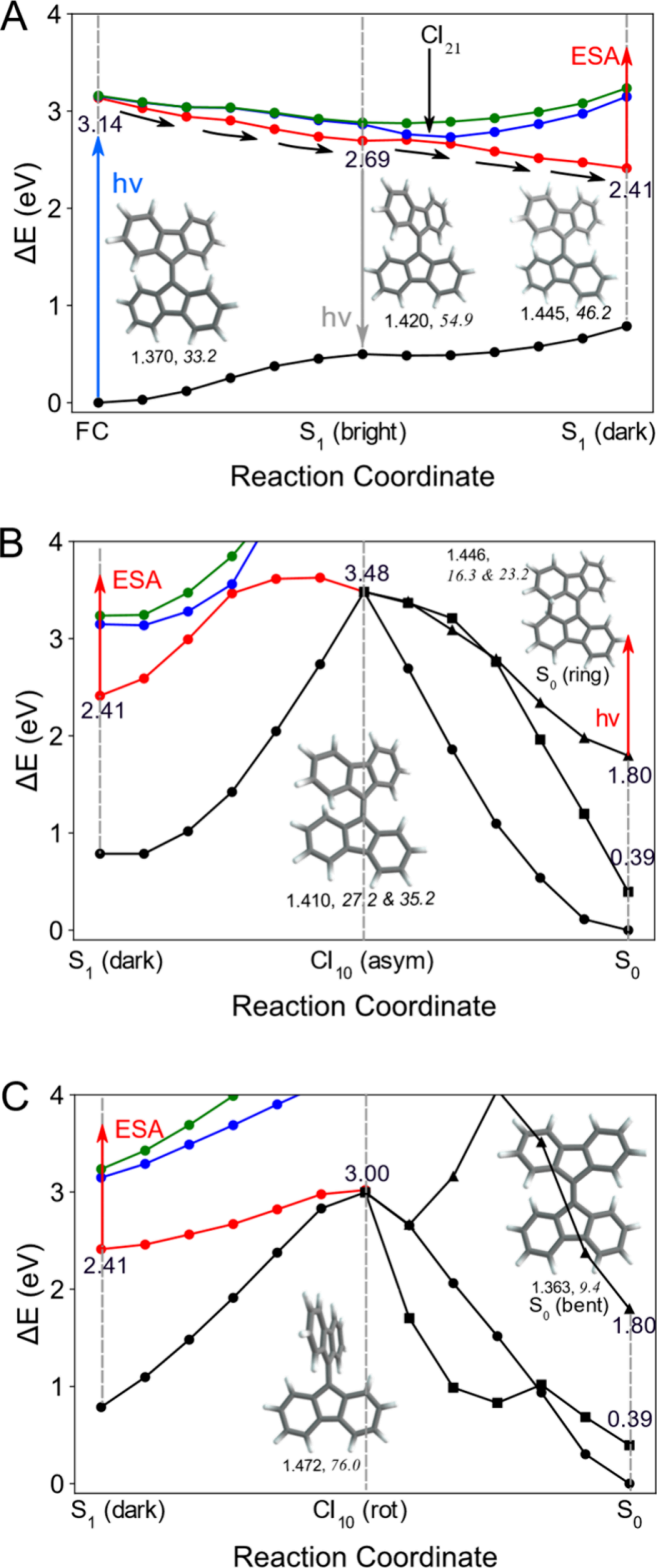
(A) Minimum-energy paths (MEPs) of the S_0_ (black line), S_1_ (red line), S_2_ (blue line), and S_3_ (green line) states connecting the FC region, S_1_ (bright), and S_1_ (dark). (B) MEPs starting from S_1_ (dark) and passing through CI_10_ (asym), leading to three possible ground-state (S_0_) conformers: S_0_ (min) (circles), S_0_ (bent) (squares), and S_0_ (ring) (triangles). (C) Same as (B), but along the pathway that proceeds via CI_10_ (rot). Optimized geometries of the key stationary points and conical intersections are shown along the reaction coordinate, along with the relative energies, the C9–C9′ distance, and the relevant dihedral angles, all in Italics. Enlarged structures with all C–C bond lengths are provided in Figure S9, and their relative energies are listed in Table S4.

The optimized molecular structures of the local minima and conical intersections are shown in Figure 4A and magnified in Figure S9. Upon excitation, the C9–C9′ bond bridging the two fluorenylidene units elongates significantly, increasing from 1.370 Å in the S_0_ minimum to 1.445 Å at the S_1_ minimum as predicted by our calculations. This drastic change in bond length in the excited-state manifold is consistent with the large resonance Raman cross-section observed for the 1551 cm^–1^ mode, which primarily corresponds to C9–C9′ stretching. Analysis of the MRSF-TDDFT excitation amplitudes shows that the S_1_ (dark) state is dominated (≈83%) by a single HOMO–1 → LUMO transition (Figure S4). In this electronic structure, the two frontier orbitals are effectively singly occupied, giving rise to pronounced diradical character at the S_1_ (dark). To further validate this picture beyond the linear-response representation, we performed Dyson orbital analyses using the Extended Koopmans’ Theorem within the MRSF-TDDFT framework.^26^ The resulting highest-occupied orbitals shown in Figure S10 clearly exhibit single occupation. The diradical character of the S_1_ state in its minimum-energy structure enhances structural flexibility on the S_1_ PES and allows increased torsional motion between the two fluorenylidene planes. This flexibility is reflected in an increase in the dihedral angle from 33.2° at the ground-state minimum to 46.2° in the optimized dark S_1_ structure. This geometric change is also reflected in several strong Raman bands in the low-frequency region, including 781, 476, and 298 cm^–1^, which correspond to torsional modes. To elucidate the accessible relaxation pathways, PESs connecting the transient dark S_1_ minimum to the ground-state configurations were mapped in Figure 4B and 4 **C**. In addition to the original ground-state minimum (S_0_ (min)), two additional ground-state structures, denoted S_0_ (bent) and S_0_ (ring), were identified together with two S_1_/S_0_ conical intersections, CI_10_ (asym) and CI_10_ (rot). In the S_0_ (bent) structure, the two fluorenylidene planes adopt a mutually curved configuration, whereas formation of a single C8–C8′ bond characterizes the S_0_ (ring) structure.

As shown in Figure 4B, a barrier of approximately 1.0 eV separates the dark S_1_ minimum from CI_10_ (asym), allowing the system to remain temporarily trapped in the excited state before undergoing a nonadiabatic transition to the ground state. Once this barrier is surmounted, all three ground-state structures—S_0_ (min) (solid circle), S_0_ (bent) (solid rectangle), and S_0_ (ring) (solid triangle)—can be accessed directly from CI_10_ (asym) without additional barriers. On the other hand, a similar early trapping in the S_1_(dark) minimum is also seen with the PES along CI_10_ (rot) due to a slightly reduced energy barrier of 0.6 eV (Figure 4C). Since the path from the S_1_ (dark) to CI_10_ (rot) requires a pronounced change in the dihedral angle between the two fluorenylidene motifs, the barrier on the PES is expected to strongly depend on solvent viscosity, as observed in many photochemical molecular motors, owing to the substantial conformational rearrangements involved.^27–30^ After the initial barrier of CI_10_ (rot) is overcome, this path predominantly favors formation of the S_0_ (min) and S_0_ (bent) structures, but not the S_0_ (ring), as the PES leading to S_0_ (ring) involves a markedly higher barrier due to the 76° large dihedral angle about the C9–C9′ bond at the CI_10_ (rot) prevents the formation of the C8–C8′ bond.

It should be noted that the absolute heights of the reaction barriers between S_1_ (dark) and the two CI_10_ are affected by many factors, such as solvent interactions, and the calculated values may vary when a different electronic-structure method is applied. Therefore, the current results should not be regarded as an accurate quantitative comparison of the two barriers. Nevertheless, our calculations consistently captured these energy barriers, which are expected to trap the molecule in its S_1_ (dark) minimum temporarily and influence the rate constant across different relaxation pathways. In this sense, the present results provide a robust qualitative picture of the relaxation landscape, even though the exact energetic values may be refined by improved theoretical treatments and by including a precise modeling of environmental effects.

The relaxation PESs outlined by the MRSF-TDDFT calculation are consistent with our experimental data and previous observations. First, experimentally observed subtle kinetic differences across different excitation energies support the calculation result that the initially observed transient species is directly related to the Franck–Condon state, rather than to a separate electronic state below the FC state, as previous studies suggested.^19^ When pumped at 420 nm rather than 475 nm, the additional 2760 cm^–1^ excitation energy can promote the molecule to a higher vibronic state on the S_1_ PES, making it easier to access the CI_10_’s. The MRSF-TDDFT calculation predicts an optical transition for the S_1_ (min) state at 620 nm, which qualitatively agrees with the observed ESA by the TA (Figure 5A), further suggesting that the S_1_ manifold was accessed directly via pump excitation. Second, the observed weak dependence of the S_1_ lifetime on solvent viscosity aligns with the presence of multiple relaxation pathways identified by the calculation. In highly viscous solvents, **BF** can funnel the excited-state energy through the CI_10_ (asym) route instead of relying on the CI_10_ (rot) route that requires large conformational changes. Third, the elongation of the lifetime of the second transient species in solvents with higher polarity implies the formation of the tilted configuration of the proposed S_0_(ring) product (Figure 4B), which has a permanent dipole moment of 1.86 D, according to calculations. If the second intermediate species include S_0_ (ring), it can be better stabilized in polar solvents, resulting in an extended lifetime. On the other hand, the fact that the lifetime of this species is not sensitive to solvent viscosity suggests that it cannot be assigned to a purely hot ground state, of which the thermalization kinetics should be influenced by solvent viscosity.

**Figure 5:**
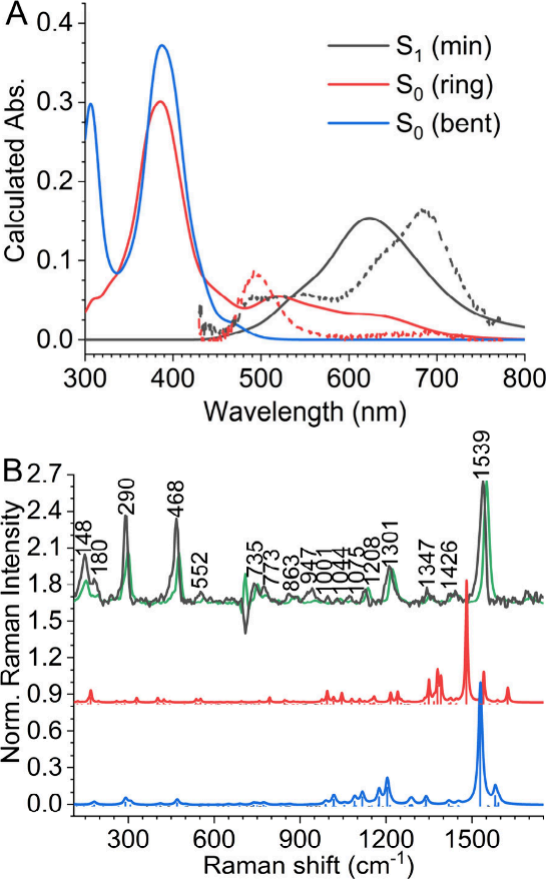
(A) Simulated absorption spectra by sampling Wigner distribution at S_1_ (min) (black), S_0_ (ring) (red), S_0_ (bent) (blue) geometries. Experimental ESA spectra reconstructed by compensating the GSB signals of spectra extracted from the global analysis, with ground state absorption, are shown as dashed lines. (B) Comparison of the FSRS spectrum collected at 10 ps time delay (black), with the calculated Raman spectra for S_0_ (ring) (red), the experimental (green) ground state, and the calculated S_0_ spectrum (blue). The calculated spectra were obtained using DFT at the B3LYP/6-311++(d,p) level and plotted with a line width of 4 cm^–1^.

TA observed no long-lived transient species surviving beyond a 150 ps time delay, implying that triplet excited states might not be significantly involved in the relaxation process. To interpret this, we conducted additional minimum energy path (MEP) calculations to include the low-lying triplet states, and the results are shown in Figure S11. Along the two major reaction pathways, T_1_ and T_2_ approach the S_1_ PES in energy at the dark S_1_ (min) configuration and near the conical intersections. However, the calculated spin–orbit couplings (SOCs) are very small, in the range of 0.025–0.31 cm^–1^ across the potential energy surfaces, suggesting a low intersystem crossing yield in the present system.

To further elucidate the relaxation products, we compared spectroscopic features observed in the TA and FSRS experiments with S_0_ (ring) and S_0_ (bent) spectra predicted by MRSF-TDDFT calculations (Figure 5). The optical transition of S_0_ (bent) is predicted in the far-UV region, beyond the spectral window of our TA experiments. In contrast, the calculation predicts that the S_0_ (ring) has an absorption band >500 nm, reproducing the ESA feature of the second transient state observed by TA. We also calculated the Raman spectrum of S_0_ (ring) and found that it reproduced the vibrational features observed in the experimental FSRS spectrum collected at a 10 ps time delay, including the overall downshifted vibrational frequencies and the spectral pattern between 800 and 1100 cm^–1^ (Figure 5B). Therefore, both TA and Raman confirm the existence of S_0_ (ring) during the relaxation. However, we cannot simply conclude that the S_0_ (ring) fully represents the second transient species because a series of Raman bands observed after a 5 ps time delay resembles bathochromically shifted ground-state signals that likely belong to the hot ground state, which also absorbs in the same ∼500 nm region.

Analysis of our comprehensive experimental and computational data leads us to conclude that the second transient signal observed in our experiment represents a mixture of the S_0_(ring) state and hot vibrational states for the minimum energy ground electronic state structure. We note that the formation of closed-ring structures could be a universal energy-dissipation route for conjugated aromatic molecules with diradical character. An earlier study on tetraphenylethylene suggested that the formation of a ring structure is responsible for its low fluorescence quantum yield in solution.^31^ Cis-stilbene also forms ring structures based on the amount of thermal energy present in its initial state.^32^ Unlike these previously reported systems, **BF** does not undergo permanent photoinduced chemical changes after illumination, as TA observed no long-lived signal beyond 300 ps. We have noticed a significant geometric distortion in the ring conformer of **BF**, resulting in a C–C bond length of 1.538 Å, significantly longer than other CC bonds in the same six-member ring, which makes the energy of S_0_ (ring) 1.80 eV higher than the S_0_ (min). Moreover, a large amount of electronic energy is rapidly transferred to vibrational levels during S_1_ relaxation, thereby thermally exciting the S_0_ (ring) structure; hence, it is easy for the C8–C8′ bond to break, returning the molecule to its original ground-state minimum. In this process, vibrationally hot ground states will also be generated, which account for the bathochromically shifted Raman signals observed in FSRS spectra.

Our findings clarify the complexity and unusual nature of the excited-state relaxation dynamics of **BF**, as summarized below. First, the low fluorescence quantum yield of **BF** was attributed to rapid relaxation from the FC state to the dark S_1_ minimum, arising from a flexible linkage between the two fluorenylidene units after photoexcitation, due to their diradical character. Second, our MRSF-TDDFT calculations identified two relaxation pathways originating from the S_1_(dark) state via different CI_10_s, ultimately yielding three ground-state relaxation intermediates. The existence of a relaxation pathway via CI_10_ (asym) that requires only minor configurational changes accounts for the weak solvent-viscosity-dependent relaxation kinetics. Finally, a vital energy-dissipation route through the formation of a transient ring structure was captured by FSRS and further supported by solvent-polarity-dependent lifetime variation of the associated transient species. This S_0_ (ring) structure is expected to quickly relax to **BF**’s ground state manifold due to the excess vibrational energy dissipated from the S_1_ (dark) state. The multiple, competing mechanistic pathways, as summarized above, also underscore the importance of carefully integrating robust time-resolved spectroscopic data with reliable electronic-structure calculations.

Results reported in this work also provide important guidelines for designing photoresponsive materials based on fluorenylidene building blocks. The flexible C9–C9′ single bond in the S_1_ state makes **BF** a potential building block for photochemical molecular motors by utilizing CI_10_ (rot) on the S_1_ PES. However, to this end, new molecular designs are needed to suppress access to CI_10_ (asym) and restrict the relative motion between the two fluorenylidene planes to controlled rotational pathways. Motors based on a **BF** motif require breaking the rotational symmetry to achieve unidirectional motions. Expanding this approach to additional macrocyclic systems based on the **BF** framework is underway in our lab, which could further deepen our understanding of how molecular architecture affects excited-state dynamics, ultimately facilitating the development of highly efficient and adaptable photonic systems.

## Supplementary Material



## Notes

Materials, NMR, TA and TSSR setup, computational methods, calculated excitation energy and frontier orbital, Raman mode assignments, TA data in different solvents and with different excitation wavelengths, optimized configurations and energies, Dyson orbital analysis, MEPs including singlet and triplet excited states, Cartesian coordinates for optimized structures. (PDF)
