# Supplementary material for: Complex and Unusual Excited-State Relaxation Dynamics of 9,9′-Bifluorenylidene Revealed by Comprehensive Time-Resolved Spectroscopy and MRSF-TDDFT Calculations
Source: J Phys Chem Lett. 2026 Mar 24;17(14):4153–60. doi: 10.1021/acs.jpclett.6c00465 (PMC13071911; doi:10.1021/acs.jpclett.6c00465)
Supplement: Supplementary file 1 [file jz6c00465_si_001.pdf]

Supporting Information

Complex and Unusual Excited-State Relaxation Dynamics of 9,9′-Bifluorenylidene Revealed by Comprehensive Time-resolved Spectroscopy and MRSF-TDDFT Calculations

Chen Wang[1,2,#,*], Woojin Park[3,#], Cheol Ho Choi[3,*], Seogjoo J. Jang[1,2], Sri Harsha Mamillapalli[4], Jinjia Xu[4,*]

Authors' address

[1]*Department of Chemistry and Biochemistry, Queens College, City University of New York, Queens, New York 11367, United States*

[2]*The Graduate Center, City University of New York, New York, New York 10016, United States*

[3]*Department of Chemistry, Kyungpook National University, Daegu 41566, South Korea*

[4]*Department of Chemistry and Biochemistry, University of Missouri−St. Louis, St. Louis, Missouri 63121, United States*

[#] C. W. and W. P. contributed equally

*Email: chen.wang@qc.cuny.edu

*Email: cchoi@knu.ac.kr

*Email: jxu@umsl.edu

Materials and Methods

All reagents and chemicals used in this study were purchased from Sigma Aldrich Chemical Co. and used as received without further purification. The solvents used for photophysical studies were of spectroscopic grade, ensuring minimal impurities and optimal performance in spectroscopic measurements. The selection of high-purity solvents was crucial for obtaining reliable and reproducible data in both steady-state and time-resolved studies. 9,9′-bifluorenylidene (**BF**) was synthesized based on our recently published paper.[1]

Nuclear Magnetic Resonance (NMR) Spectroscopy: NMR spectra were recorded on a Bruker Avance 300 (300 MHz) spectrometer. All chemical shifts were calibrated against tetramethylsilane (TMS) as the internal standard. The spectra provided detailed structural

information about the synthesized compounds, confirming the chemical integrity and purity of the sample. Peak assignments and coupling constants were analysed to identify the electronic environments of key functional groups in 9,9′-bifluorenylidene (**BF**).

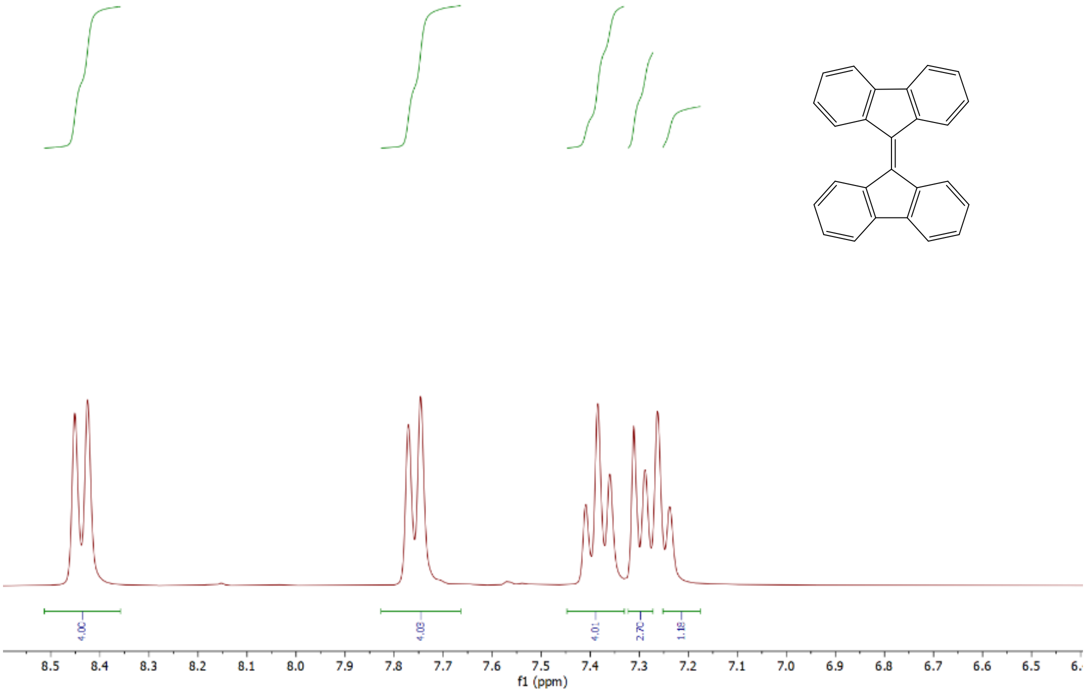

Transient Absorption (TA) Spectroscopy: TA spectroscopy was conducted using a homemade system described in detail in a previous publication.[2] Briefly, 800 nm, 150 fs pulses, generated from a pulse-regenerated amplifier (Uptek, Regen) at 1000 Hz, were employed as the fundamental to power the system. About 1.5 W of the 800 nm fundamental was delivered to an optical parametric amplifier (Light Conversion, TOPAS) to generate signal and idler pulses at different wavelengths for producing pump excitation. The 420 nm pump excitation was generated with the fourth-harmonic generation of the 1680 nm idler using two BBO crystals. The 475 nm pump excitation was generated with the sum-frequency generation of the 1170 nm signal and the 800 nm fundamental using a BBO crystal. Short-pass filters were used to remove the IR residual from the pump beams. A small portion of the 800 nm fundamental was diverged to generate a broadband white light probe with a CaF_2 crystal. After filtering the

800 nm light with a short-pass filter, the probe covers the spectral range from 430 to 770 nm. The probe beam was divided into two arms: one to overlap with the 420 nm pump beam at the sample to probe the TA signal, and the other to be used as the reference beam to cancel the pulse-to-pulse fluctuation. The two probe arms were separated vertically by 2.4 cm at the sample plane. The pump and probe were diverged by 6º before they were focused to sample using different lenses. After the sample, the two probe beams were collimated parallelly and focused on the slit of the spectrometer (SpectraPro-300i, Acton). A 300 groove/mm grating dispersed the signal and reference arms into two vertically aligned photodiode arrays (EB Stressing). TA signals were calculated as $\Delta OD = -\log(\left(\frac{I_S}{I_R}\right)_{pump-on} / \left(\frac{I_S}{I_R}\right)_{pump-off})$. The time delay of the system was generated by optically delaying the incoming probe pulse with a retroreflector installed on a translation stage. The instrument response function (IRF), which was approximately 200 fs, sets the effective time resolution of the experiment. Four solvents were explored to study the effects of solvent viscosity and polarity on relaxation dynamics. Solvent properties are listed in **Table 2**. Global analysis of the TA data was conducted using a MATLAB toolbox developed by van Wilderen et al.[3]

Femtosecond Stimulated Raman Spectroscopy (FSRS): FSRS experiments were performed using a homemade system built atop the TA setup. The actinic pump and the Raman probe used for the experiments come from the same source as the TA pump and probe. The Raman pump pulse was generated by sending half of the 800 nm, Regen output energy to a specialized doubling/bandwidth-narrowing unit (Light Conversion, SHBC). The SHBC generated 400 nm pulses with a bandwidth <10 cm^{-1} and a pulse duration of ~2 ps. The 400 nm pulse was focused by a 300 mm focal-length lens into a Raman shifter consisting of a 0.5 m pipe filled with 300 psi CH_4**.** The vibrational frequency of CH_4 (2917 cm^{-1}) yields the first- and second-Stokes stimulated Raman lines at 453 and 522 nm. The second Stokes Raman line was picked up with a Perlin Broca prism. A waveplate and polarizer combination were employed to adjust the Raman pump energy.

The 420 nm actinic pump and the 522 nm Raman pump were chopped by two optical choppers at 250 Hz. The phases of the two choppers were offset by 90° to generate an on-off pattern, as shown in **Figure S1**. The two pump beams were combined colinearly at a 50/50 beam splitter and focused on the sample with a concave mirror (f = 150 mm). The WL Raman probe was focused by a separated concave mirror and overlapped with the actinic/Raman pump at the sample. The Raman probe and the two pump beams were diverged by 6º. Only the Raman probe was sent to the spectrograph slit, where a 1200 gr/mm grating dispersed the spectrum onto the photodiode array detector. As shown in **Figure S1**, Raman probe signals were binned separately according to the on/off actinic and Raman pump illuminations: $I_{on\text{-}on}$, $I_{on\text{-}off}$, $I_{off\text{-}off}$, and $I_{off\text{-}on}$. With these combinations, several signals can be measured (**Figure S2**): 1) the non-pumped Raman gain was

obtained as $RG_{no\ pump} = \frac{I_{off-on}}{I_{off-off}}$; 2) Raman gain difference caused by the actinic pump was generated as $RG_{diff} = \frac{I_{on-on}}{I_{on-off}} - \frac{I_{off-on}}{I_{off-off}}$; 3) a transient absorption signal, $TA = \frac{I_{on-off}}{I_{ff-off}}$, was collected to analyze the spectral background. The solvent-only spectrum was collected separately to remove the solvent Raman signals.

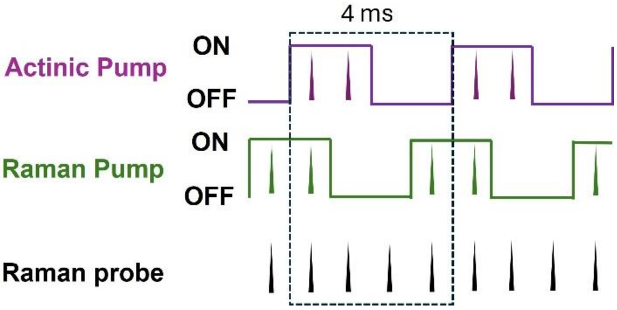

Figure S1. Pulse train of the FSRS experiment with offset chopped actinic pump and Raman pump pulses. A 4 ms time box (dashed line) was placed to indicate pulses included in one experimental cycle.

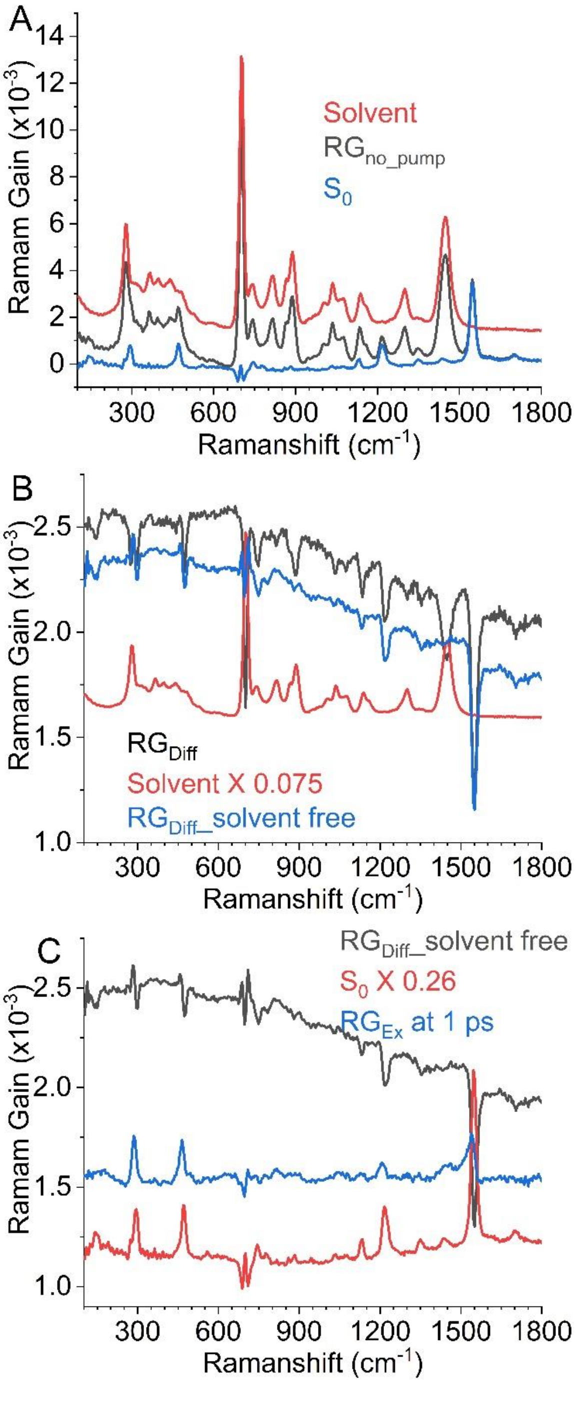

Figure S2. Data workup for the FSRS experiment of **BF** in hexane/DCM (5:1) at 1 ps time delay after the actinic pump. **(A)** The ground-state Raman spectrum was obtained by subtracting the scaled solvent spectrum (×0.93) **(B)** Negative signals from the solvent were removed from the RG_{Diff} by adding a scaled solvent spectrum (×0.075). **(C)** The excited-state spectrum was obtained by adding the ground-state Raman spectrum scaled by 0.26 to remove the negative ground-state features.

The pulse energy of the actinic pump excitation was 0.1 µJ, and the pulse energy of the Raman pump was 0.6 µJ. Temporal and spatial overlap of the Raman pump and Raman

probe was determined by maximizing the stimulated Raman signal of the solvents. The overlap of the actinic pump and the Raman probe was determined by maximizing the TA signal. An optical delay stage was employed to adjust the arrival time of the actinic pump. Raman shifts of the spectra were calibrated by the toluene Raman signals as shown in **Figure S3**.

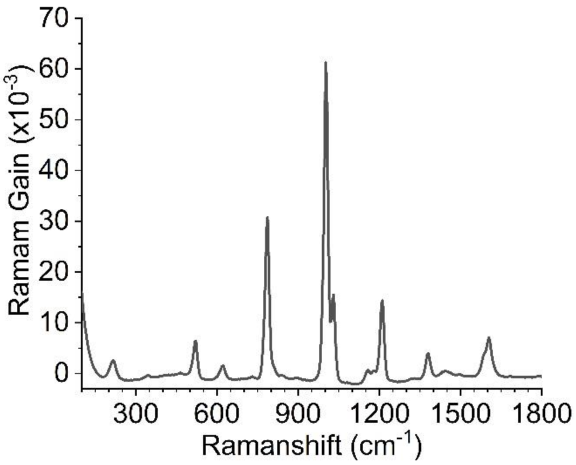

Figure S3. Stimulated Raman signal of toluene collected for calibrating the system's Raman shifts.

Computational Methods.

Considering the large system size, conventional wavefunction-based multireference methods are computationally prohibitive. Therefore, density functional theory (DFT) and its time-dependent extension (TDDFT) can be employed as practical alternatives. However, standard DFT and TDDFT fail to describe regions near the conical intersection (CI) between the ground and first excited states. To overcome this limitation, we employed the mixed-reference spin-flip time-dependent density functional theory (MRSF-TDDFT),[1] in combination with the BH&HLYP functional and 6-31G(d) basis set. The ground- and excited-state geometry optimizations and the search for CIs were performed using MRSF-TDDFT. Minimum energy paths (MEPs) are generated by geodesic interpolation between the optimized geometries.[2] The MRSF-TDDFT calculations were done in the GAMESS-US package.[3] The MRSF-TDDFT incorporates static multi-configurational (e.g., double-excitation) descriptions and dynamic electron correlation within the linear-response framework, thereby providing a balanced and computationally efficient description of both ground and excited states. A recent perspective further underscores the merits of MRSF-TDDFT in this context.[4, 5] The ground- and excited-state absorption spectra were constructed by obtaining vertical excitation energies and oscillator strengths using 100 structures from the Wigner distribution of the ground- and excited-state equilibrium geometries of **BF** at 300K. The Wigner distributions were constructed within the harmonic approximation using the normal modes and vibrational frequencies at each equilibrium geometry. Each vertical transition was convoluted with a Gaussian function (σ = 10 nm) to account for inhomogeneous broadening, and the total absorption spectrum was obtained by

summing over all broadened transitions. The number of sampled geometries was chosen to ensure convergence of the spectral profile. To obtain ground-state properties, including Raman and IR spectra, additional calculations were performed. Geometry optimization and the Raman spectra of **BF** and the ring conformer of **BF** were calculated using DFT at B3LYP/6-311++G(d,p) level. Before the final geometry optimization, the ground-state molecular structures were first optimized using the Hartree-Fock method with the 3-21G basis set and further optimized using DFT at B3LYP/6-31G(d) level. The normal-mode frequencies of the ground-state spectra were scaled by a factor of 0.9679.[6] Normal mode diagrams prepared using Jmol are illustrated in **Table S2**.[7]

Table S1. Vertical excitation energies (VEEs) of the lowest singlet excited states of **BF** obtained by MRSF-TDDFT theory with various basis sets and functionals. Excitation energies are given in electron volt (eV). Oscillator strengths and major electron configurations are given in parentheses. The BH&HLYP/6-31G(d) optimized S_0 structure was used in all calculations.

	S_1	S_2	S_3
BH&HLYP/6-31G(d)	3.136 (0.8630) (H → L)	3.152 (0.0022) (H-1 → L)	3.159 (0.0001) (H-2 → L)
BH&HLYP/cc-pVTZ	3.055 (0.8429) (H → L)	3.103 (0.0018) (H-1 → L)	3.107 (0.0002) (H-2 → L)
CAMB3LYP/6-31G(d)	2.910 (0.8391) (H → L)	2.932 (0.0021) (H-1 → L)	2.940 (0.0002) (H-2 → L)
CAMB3LYP/cc-pVTZ	2.845 (0.8184) (H → L)	2.892 (0.0022) (H-1 → L)	2.899 (0.0002) (H-2 → L)
B3LYP/6-31G(d)	2.304 (0.8669) (H → L)	2.471 (0.0001) (H-1 → L)	2.474 (0.0001) (H-2 → L)
B3LYP/cc-pVTZ	2.257 (0.8483) (H → L)	2.442 (0.0000) (H-1 → L)	2.442 (0.0001) (H-2 → L)

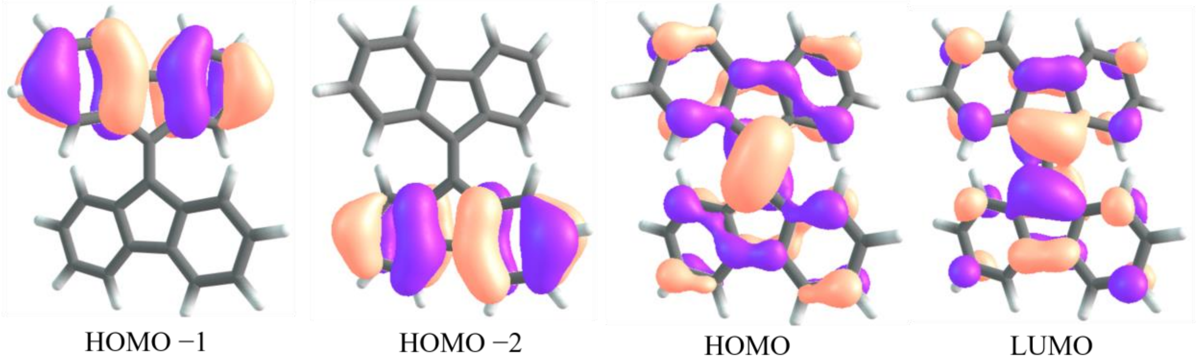

Figure S4. Frontier molecular orbitals (H–2 to L) of BF contributing to the S_1–S_3 excitations calculated at the MRSF-TDDFT level.

Table S2 Raman mode assignments for **BF** based on DFT calculation (B3LYP/6-311++G(d, p)). Calculated frequencies are provided in parentheses.

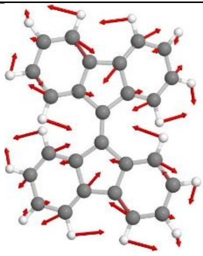 1608 (1580) cm^{-1}	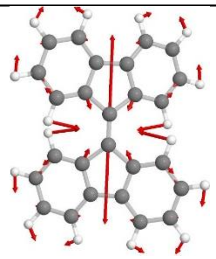 1551 (1528) cm^{-1}	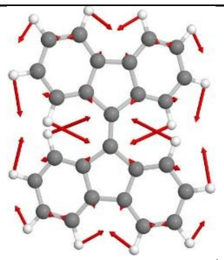 1444 (1418) cm^{-1}
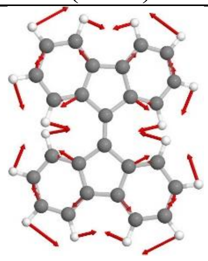 1352 (1339) cm^{-1}	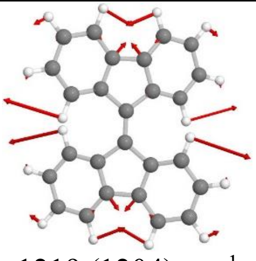 1219 (1204) cm^{-1}	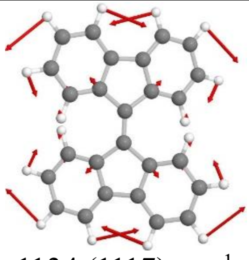 1134 (1117) cm^{-1}
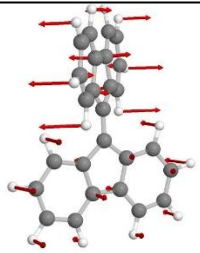 781 (772) cm^{-1}	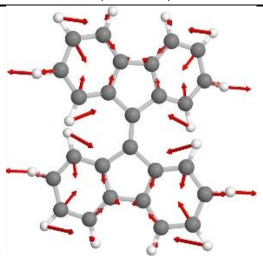 751 (750) cm^{-1}	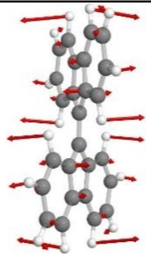 476 (470) cm^{-1}
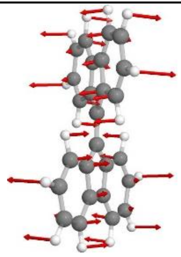 298 (291) cm^{-1}	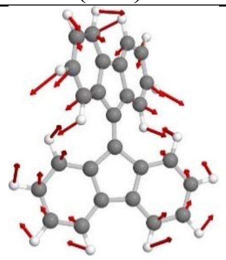 152 (182) cm^{-1}	

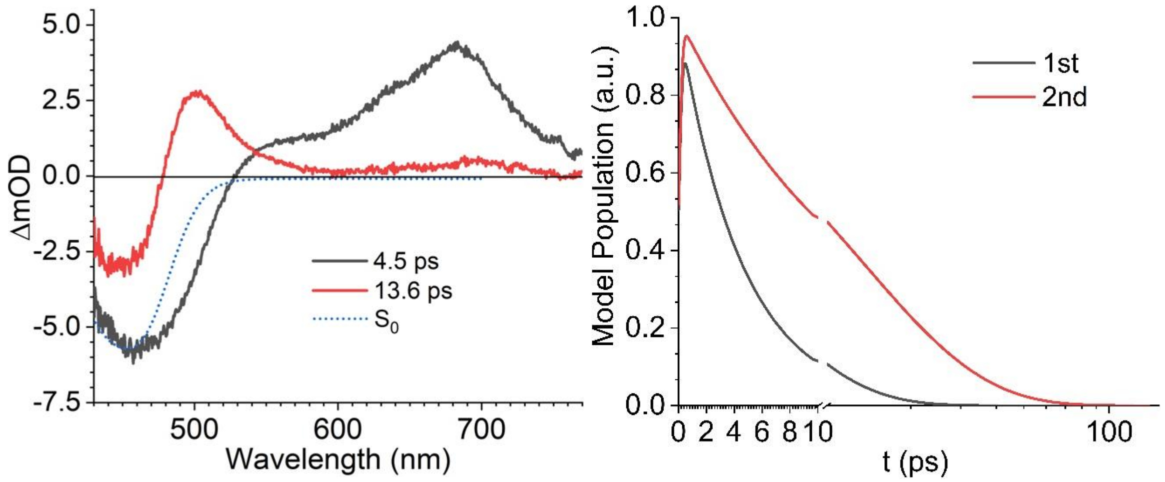

Figure S5. Spectra and population kinetics obtained from global analysis of the fs-TA data of **BF** in hexanes using a model involving two parallel decay pathways. The excitation wavelength was 420 nm. A reserved ground state absorption spectrum was scaled to match the minimum amplitude of the negative signal in the 4.5 ps spectrum for comparison. We found that the negative signal in the 4.5 ps spectrum is significantly broader than the ground-state absorption range, suggesting that the spectrum extracted from the parallel model cannot be attributed to a specific state.

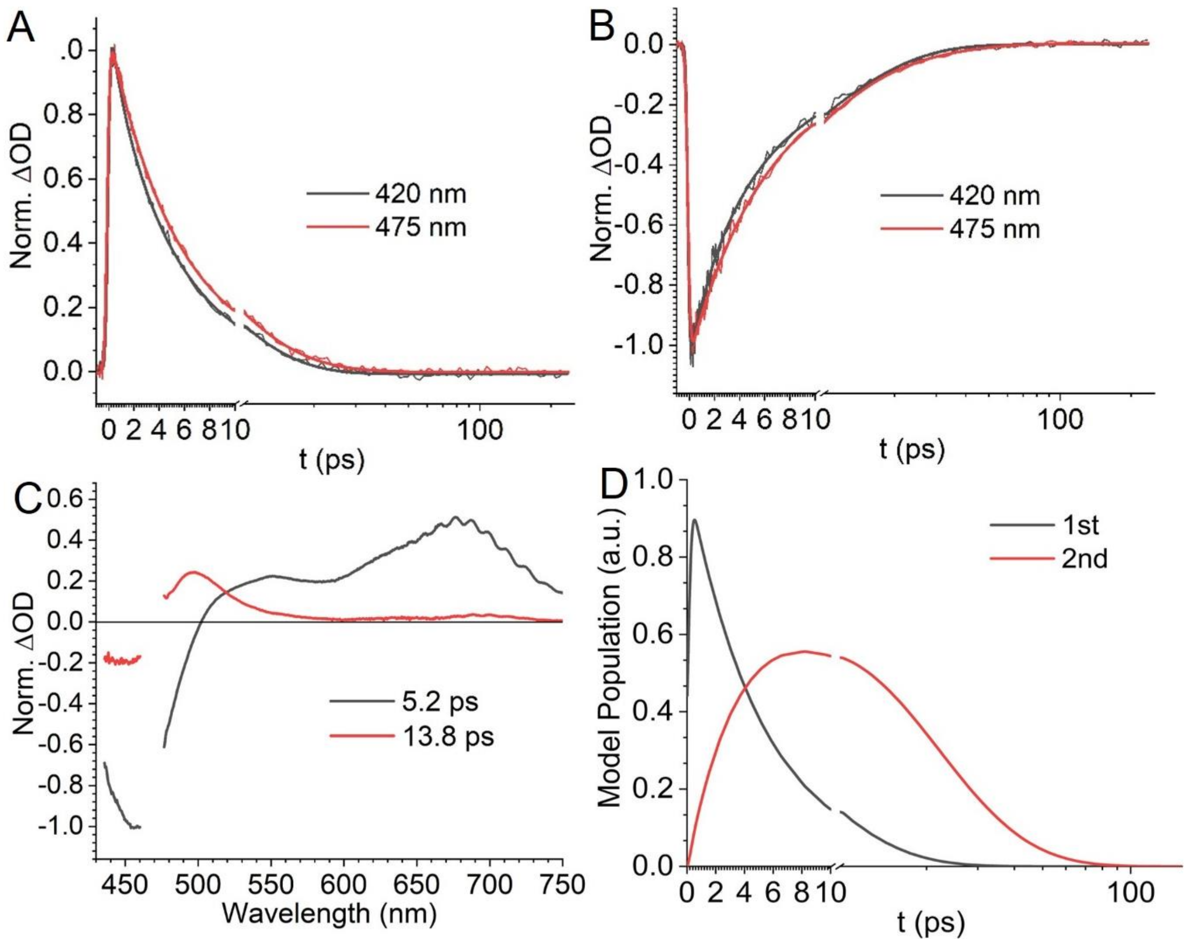

Figure S6. Comparison of normalized TA kinetics of **BF** in n-hexane with 420 nm and 475 nm pump at **(A)** ESA at 680 nm and **(B)** GSB at 454 nm. Exponential fitting parameters are listed in **Table S3. (C)** Species-associated spectra and **(D)** the kinetics of transient populations extracted from global analysis of the fs-TA data with a three-state, sequential decay model. The spectra were scaled by normalizing the GSB minimum to -1.0. The blank in **(C)** corresponded to the spectral region affected by scattering from the 475 nm pump and was trimmed before global fitting.

Table S3. Comparison of kinetic parameters for fitting traces in **Figure S6A** and **B**.

Signals (nm)	Pump (nm)	τ_1 (ps), A_1 (%)	τ_2 (ps), A_2%
ESA 680	420	4.9 ± 0.1,	
	475	5.7 ± 0.1	
GSB 454	420	4.5 ± 0.8, -64 ± 11	13 ± 3, -36 ± 11
	475	5.2 ± 0.7, -69 ± 5	14 ± 2, -31 ± 4

*Errors were generated from the kinetic fitting.

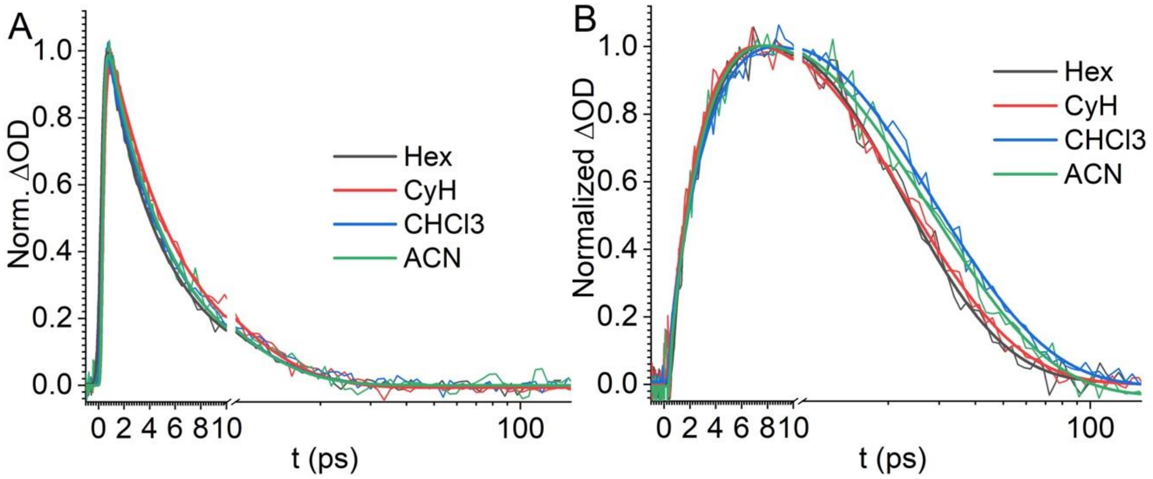

Figure S7. Comparison of normalized TA kinetics of **BF** in different solvents. The selected kinetic traces were extracted at **(A)** ESA around 680 nm, and **(B)** ESA around 505 nm. The lifetime of the species corresponding to the 680 nm ESA increases with solvent viscosity, whereas the latter species corresponding to the 505 nm ESA lasts longer in polar solvents.

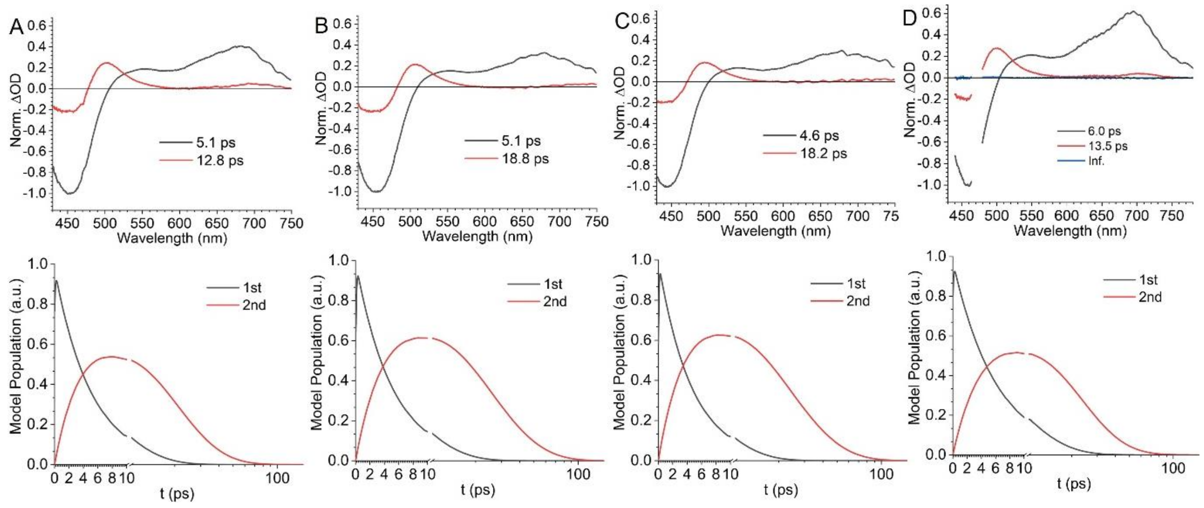

Figure S8. Species-associated spectra (upper) and the kinetics of transient populations (lower) extracted from global analysis of the fs-TA data with a three-state, sequential decay model for **BF** in **(A)** cyclohexane, **(B)** $CHCl_3$, **(C)** acetonitrile with 420 nm excitation, and **(D)** in cyclohexane with 475 nm excitation. Lifetimes of the two transient species are indicated in the legend. The spectra were scaled by normalizing the GSB minimum of the first transient species to -1.0.

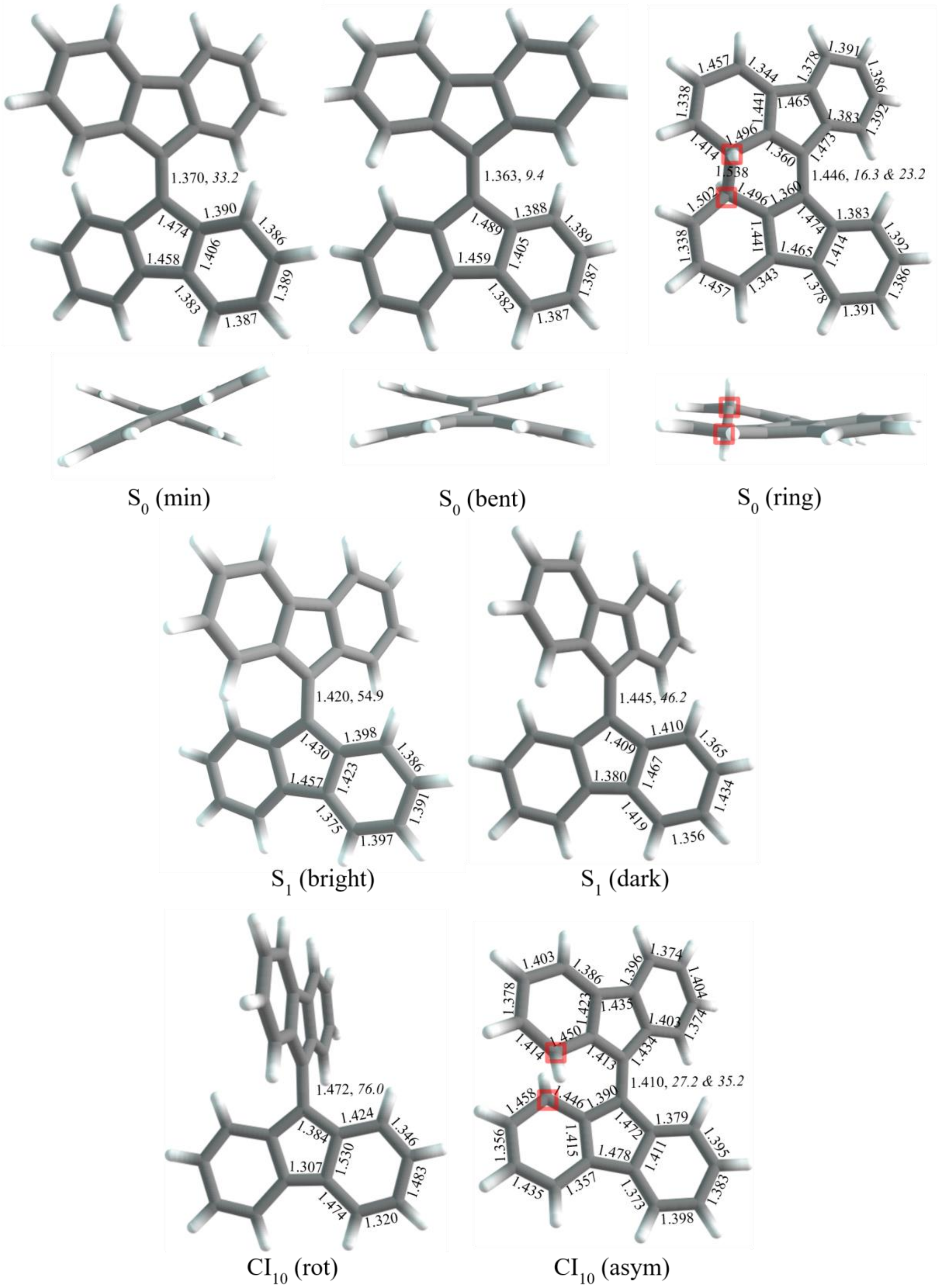

Figure S9. Optimized geometries of the ground (S_0), excited (S_1), and conical intersection (CI) structures. Bond lengths are shown in Angstrom, and the C9– C9′ torsion angles are given in degrees in Italic. For nearly symmetric structures, only unique parameters are presented. The C8 and C8″ carbons in CI_{10}(asym) and the relaxation product S_0(ring) are marked in a red box.

Table S4. The energies (in eV) of optimized minima and conical intersections relative to the S_0 (min) state. See **Figure. S9** for structures.

	S_0	S_1	S_2
S_0 (min)	0.00	3.13	3.15
S_0 (bent)	0.39	3.93	3.96
S_0 (ring)	1.80	4.25	4.78
S_1 (bright)	0.50	2.69	2.86
S_1 (dark)	0.79	2.41	3.15
CI_{10} (asym)	3.48	3.48	4.34
CI_{10} (rot)	3.00	3.00	4.19

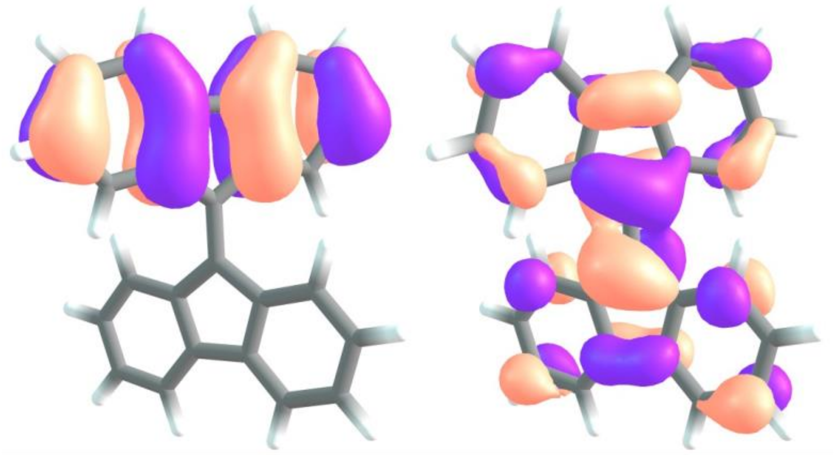

HOMO −1
(0.51)

LUMO
(0.58)

Figure S10. Dyson orbital of the S_1 state of **BF** calculated using MRSF-TDDFT/BH&HLYP/6-31G(d) at the S_1 (dark) optimized structure. The respective norms are given in parentheses.

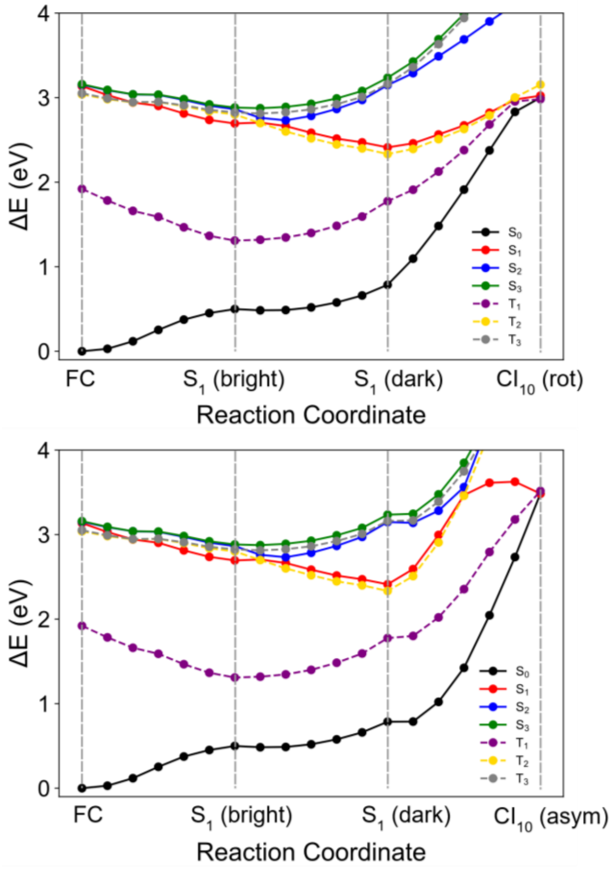

Figure S11. Minimum-energy paths (MEPs) including both singlet and triplet states, connecting the Franck–Condon (FC) region, S_1 (bright), S_1 (dark), and the CI_{10} structures along the rotational (upper panel) and asymmetric (lower panel) pathways.

Cartesian Coordinates optimized at MRSF-TDDFT/BH&HLYP/6-31G(d) level of theory

S_0 (min)

C -5.0003426258 2.6310231935 1.1694231779
C -6.0161502698 1.8293986348 0.6636958964
C -5.7248267145 0.6783033813 -0.0518284856
C -4.3981295633 0.3400968696 -0.2942953116
C -3.3803474736 1.1259182256 0.2752627050
C -3.6730009905 2.2715385488 0.9916134154
C -2.1001066410 0.4804845891 0.0104797454
C -2.3460306003 -0.7312699292 -0.6594461893
C -3.7953688428 -0.8646084655 -0.8925599476
C -0.8114465318 0.9089844469 0.2696300617
C 0.2536071749 0.1354699318 -0.1664288079
C 0.0229919436 -1.0328021065 -0.8822725826
C -1.2681482476 -1.4625115253 -1.1461314295
C -4.4424155476 -1.8888983709 -1.5322283855
C -5.6595426277 -1.8011664902 -2.3589542896

C	-5.9698142690	-3.0810744971	-2.8525623275
C	-5.0032521530	-4.0286369104	-2.3107142866
C	-4.0657764915	-3.3137344586	-1.5442213132
C	-6.3614129952	-0.7039096055	-2.8452270702
C	-7.4084147826	-0.9015176249	-3.7320528889
C	-7.7436599169	-2.1778764998	-4.1668499303
C	-7.0113835670	-3.2755153886	-3.7406030164
C	-3.1239841195	-4.0181404696	-0.8026592605
C	-3.0684402405	-5.3993030555	-0.9056933825
C	-3.9614422949	-6.0903324387	-1.7155146753
C	-4.9472953655	-5.4064363660	-2.4106975440
H	-7.0455781731	2.0972462996	0.8380624327
H	-6.5278687649	0.0535304497	-0.3991432402
H	-2.8845767245	2.8668935691	1.4236033797
H	-5.2458728633	3.5221837620	1.7239637617
H	-0.6335352802	1.8396147184	0.7844481049
H	1.2645126918	0.4547397922	0.0287279984
H	0.8574452925	-1.6086846613	-1.2481856703
H	-1.4217151633	-2.3485723776	-1.7353114060
H	-6.0959145355	0.2963837757	-2.5541071309
H	-7.9606374842	-0.0504335298	-4.0961300929
H	-8.5599481779	-2.3125748151	-4.8578300960
H	-7.2408395907	-4.2613002735	-4.1126335399
H	-2.4476088925	-3.5079004732	-0.1408311802
H	-2.3290992036	-5.9428893806	-0.3402565051
H	-5.6701906738	-5.9451990002	-3.0022609444
H	-3.9035997102	-7.1645809687	-1.7831851750

S_0 (bent)

C	0.0384684362	-2.0619683186	1.4263951134
C	-0.1225985824	-3.3982247799	1.0921409756
C	-1.1897491052	-3.8140852297	0.3066380761
C	-2.1197491251	-2.8862588264	-0.1403364847
C	-1.8736977007	-1.5243547298	0.1059135031
C	-0.8283967102	-1.1105825425	0.9094175990
C	-2.7480074941	-0.7379389687	-0.7577174028
C	-3.5232701038	-1.6246626342	-1.5247329244
C	-3.3394725203	-3.0015206984	-0.9869267954
C	-2.7546721384	0.6230162009	-0.9974257392
C	-3.4898558684	1.1127619082	-2.0665497881
C	-4.1623891194	0.2349281269	-2.9036758855
C	-4.1768604721	-1.1292655599	-2.6438957477
C	-4.1863011353	-4.0634819802	-1.0982640469
C	-4.0025746788	-5.4436027694	-0.5689615227
C	-4.7818294636	-6.3245770276	-1.3391285265
C	-5.6607538653	-5.5316316100	-2.1921496075
C	-5.4098238046	-4.1719962483	-1.9400364109
C	-3.3439519933	-5.9479946181	0.5432127761
C	-3.3564178173	-7.3144449410	0.7918731351

C	-4.0320500436	-8.1857358593	-0.0494747574
C	-4.7729172922	-7.6874730548	-1.1108510404
C	-6.3408092437	-3.2390230661	-2.3739153024
C	-7.4158238054	-3.6476052902	-3.1523037440
C	-7.5832627541	-4.9816927812	-3.4922320761
C	-6.7142036315	-5.9381395513	-2.9888607450
H	0.5955276595	-4.1261023442	1.4336606805
H	-1.2563103955	-4.8466626735	0.0226639584
H	-0.6635589912	-0.0622692603	1.1004447796
H	0.8643992318	-1.7574930942	2.0485444223
H	-2.1663279557	1.2909988907	-0.3889023647
H	-3.4999473296	2.1704524076	-2.2742343007
H	-4.6715389005	0.6106696525	-3.7762837821
H	-4.6632384701	-1.7876374213	-3.3378875257
H	-2.8561195996	-5.2952099741	1.2412655732
H	-2.8433734458	-7.6970078908	1.6592441002
H	-4.0197662830	-9.2451793329	0.1489084130
H	-5.3636374125	-8.3506771507	-1.7222403332
H	-6.2678198951	-2.2078995311	-2.0857323698
H	-8.1348639733	-2.9159084407	-3.4833710202
H	-6.8837052534	-6.9849156427	-3.1841612914
H	-8.4157484555	-5.2805025128	-4.1083719641

S_0 (ring)

C	-1.7593035401	-3.3579343390	3.5618370453
C	-1.2226100540	-3.5259341984	2.3479503584
C	-1.8291742239	-2.9294448631	1.1096958487
C	-3.2083805647	-2.4198521873	1.3877864358
C	-3.7274000980	-2.2364477342	2.7191215345
C	-3.0285668563	-2.6772209912	3.7786263850
C	-5.0797992167	-1.6973474565	2.5536221469
C	-5.3443552492	-1.6338048239	1.1663321641
C	-4.1359624620	-2.0810259558	0.4520621268
C	-6.0408447602	-1.3454163735	3.4770066056
C	-7.2893916089	-0.9302513350	3.0255780373
C	-7.5647029269	-0.8966911343	1.6679271519
C	-6.6008375436	-1.2549609450	0.7302415915
C	-3.7690430249	-2.2846575499	-0.9320804679
C	-2.7025922066	-3.0949586083	-1.1692971226
C	-2.4079814258	-3.1612624016	-2.5778243078
C	-3.4249422226	-2.3414157720	-3.2414951759
C	-4.2376765610	-1.7749330821	-2.2328834061
C	-1.9134322849	-3.8316892007	-0.1332177550
C	-0.6176197193	-4.3308431619	-0.7068898049
C	-0.3728411638	-4.3406112746	-2.0220937053
C	-1.2890684170	-3.7721628208	-3.0014905192
C	-5.2190971099	-0.8653143491	-2.5818154352
C	-5.4188178406	-0.5651187676	-3.9259987092
C	-4.6411093088	-1.1496794126	-4.9128105946

C	-3.6259239183	-2.0367911527	-4.5707087510
H	-0.2794706510	-4.0408743499	2.2499022623
H	-1.1970322120	-2.0723449283	0.8267314227
H	-3.4070694036	-2.5934116395	4.7848900857
H	-1.2527502264	-3.7571366303	4.4263729898
H	-5.8345362212	-1.3954474719	4.5346824652
H	-8.0491049749	-0.6466321447	3.7355014308
H	-8.5429804209	-0.5946553978	1.3299329816
H	-6.8490364398	-1.2558848052	-0.3164911123
H	-2.4910306478	-4.7207007358	0.1678931209
H	0.1010111175	-4.7639196778	-0.0285219846
H	0.5492534646	-4.7638823512	-2.3881410472
H	-1.0180618548	-3.8040357500	-4.0447860726
H	-5.8119783002	-0.3722913845	-1.8315205441
H	-6.1883761448	0.1381602121	-4.2009453421
H	-3.0039123046	-2.4731129501	-5.3364815070
H	-4.8143873378	-0.9061868251	-5.9483424092

S_1 (bright)

C	-5.0610044928	2.4030588225	1.4225977903
C	-6.0535355153	1.5482668162	0.9565090643
C	-5.7332494687	0.4396018711	0.1882099858
C	-4.3915229358	0.2009492961	-0.1224307096
C	-3.3786534649	1.0692163809	0.3732480729
C	-3.7153782821	2.1637710339	1.1344034644
C	-2.0989877924	0.5458074859	-0.0847281833
C	-2.3708006870	-0.6220914165	-0.8512293439
C	-3.7823298466	-0.8457859293	-0.881875342
C	-0.8036409186	0.9706063834	0.0953856470
C	0.2367499594	0.2469290303	-0.4918084748
C	-0.0238441392	-0.8855818825	-1.2553875349
C	-1.3231042723	-1.3281203340	-1.4492362689
C	-4.4530361523	-1.9081326819	-1.5446638079
C	-5.4986132718	-1.7798349808	-2.5109037095
C	-5.9022251155	-3.0760515337	-2.9383134677
C	-5.0806699844	-4.0419107820	-2.2214127490
C	-4.2061953693	-3.3057975442	-1.3738879341
C	-6.0982459240	-0.6421851814	-3.0584133628
C	-7.1069546759	-0.8048737205	-3.9951120844
C	-7.5094504570	-2.0741133709	-4.3956137643
C	-6.9041588544	-3.2177239866	-3.8693662579
C	-3.3137271557	-3.9774994958	-0.5337262329
C	-3.2794684640	-5.3629680043	-0.5672884193
C	-4.1237143497	-6.0763858113	-1.4109960726
C	-5.0322082171	-5.4160216001	-2.2413539834
H	-7.0842533233	1.7467848957	1.2013742268
H	-6.5027768522	-0.2280045799	-0.1610335667
H	-2.9573614017	2.8333808524	1.5094470113
H	-5.3312546109	3.2593118643	2.0187418462

H	-0.5843033720	1.8503814682	0.6797869559
H	1.2542293148	0.5746250075	-0.3543309098
H	0.7945462556	-1.4217491922	-1.7075428688
H	-1.5236126222	-2.1985672566	-2.0508491103
H	-5.7792773964	0.3420324457	-2.7594167528
H	-7.5808144602	0.0632849035	-4.4233880036
H	-8.2950445313	-2.1785458886	-5.1259339336
H	-7.2242051806	-4.1947781232	-4.1957434791
H	-2.6674703267	-3.4265133210	0.1285268822
H	-2.5945397300	-5.8946737056	0.0730071961
H	-5.6839640150	-5.9848997798	-2.8857189984
H	-4.0810889073	-7.1532579487	-1.4214382426

S_1 (dark)

C	-5.0214890413	2.5604618068	1.2827296131
C	-6.0258912970	1.7233449551	0.8053726232
C	-5.7171710733	0.5811093497	0.0853247667
C	-4.3811916399	0.2824689897	-0.1760542545
C	-3.3654388825	1.1214530586	0.3406486057
C	-3.6848052320	2.2563303319	1.0607097536
C	-2.0825734866	0.5370185190	-0.0238004897
C	-2.3427568552	-0.6423519280	-0.7610680431
C	-3.7708508061	-0.8263576288	-0.8710397020
C	-0.7857597694	0.9481882424	0.2163103592
C	0.2655525575	0.1921608089	-0.2852539216
C	0.0175530949	-0.9527426577	-1.0372786207
C	-1.2784419015	-1.3717633831	-1.2879484870
C	-4.4529860132	-1.9068148132	-1.5457990280
C	-5.5405758892	-1.7994672111	-2.4353696248
C	-5.9333216572	-3.1345649080	-2.9012352847
C	-5.0856478968	-4.0435768812	-2.3011791345
C	-4.1635000087	-3.2825860107	-1.4503865862
C	-6.2274778561	-0.6853613536	-2.9593548048
C	-7.2530317060	-0.8887867282	-3.8376817278
C	-7.6586652962	-2.1952633271	-4.2668164486
C	-7.0071645438	-3.2951911571	-3.8151207521
C	-3.2321175668	-4.0003881286	-0.6725093807
C	-3.1907182536	-5.3614624555	-0.7726205949
C	-4.0670168198	-6.1024263992	-1.6321137880
C	-5.0007700796	-5.4583595005	-2.3748811768
H	-7.0579834752	1.9646389533	1.0032517053
H	-6.5028816611	-0.0689204503	-0.2613951673
H	-2.9104910104	2.8980791818	1.4509710159
H	-5.2833390820	3.4463731281	1.8384405978
H	-0.5867270403	1.8452579484	0.7814780377
H	1.2819853919	0.4996677424	-0.0995678809
H	0.8456911104	-1.5176903860	-1.4342328480
H	-1.4579783857	-2.2478641164	-1.8881411212
H	-5.9462678494	0.3117299037	-2.6691722450

H	-7.7814755268	-0.0361871291	-4.2334145756
H	-8.4794205106	-2.2862386709	-4.9579774795
H	-7.2935086256	-4.2821192359	-4.1412077610
H	-2.5637781327	-3.4793926013	-0.0097622666
H	-2.4752447256	-5.9098532986	-0.1808995733
H	-5.6746182835	-6.0057548290	-3.0141008070
H	-3.9768652813	-7.1748572244	-1.6697589281

CI_{10} (rot)

C	-4.9527279645	2.1498409133	1.8715318910
C	-5.9341157262	1.2523256878	1.4540412535
C	-5.6334323741	0.2393161782	0.5611904113
C	-4.3275322002	0.1259764540	0.0785101253
C	-3.3302421481	1.0435999850	0.5125307290
C	-3.6447482787	2.0473265252	1.4024704572
C	-2.0883787708	0.6722355943	-0.1554309269
C	-2.3811856806	-0.4437448971	-0.9880835483
C	-3.7553900994	-0.7911043041	-0.8485632417
C	-0.8107722183	1.1854019020	-0.0998621127
C	0.1888046592	0.5960922685	-0.8712480169
C	-0.0914534421	-0.4952402260	-1.6919690342
C	-1.3690963832	-1.0212111880	-1.7582089400
C	-4.4528024302	-1.8922675517	-1.5327434556
C	-5.3104999892	-1.8039177796	-2.6143873001
C	-5.8117653824	-3.1908605595	-3.0222370227
C	-5.2566103046	-4.0490288646	-2.2068667640
C	-4.3805409754	-3.2436864264	-1.2454212409
C	-5.7783660316	-0.6900733469	-3.3689239057
C	-6.6346574318	-0.9071527010	-4.3848356021
C	-7.1370701217	-2.2405842207	-4.7978779735
C	-6.7471017333	-3.3248152354	-4.1533736831
C	-3.6504087176	-3.9686951010	-0.2604536150
C	-3.7635929851	-5.3097849577	-0.2243145893
C	-4.6127199896	-6.1121679593	-1.1385843216
C	-5.3229179321	-5.5158315015	-2.0782904889
H	-6.9388152434	1.3514193499	1.8328370078
H	-6.3909240867	-0.4566382979	0.2380624168
H	-2.8961181347	2.7492723221	1.7358105832
H	-5.2085720141	2.9342047248	2.5655388390
H	-0.5780700598	2.0299184328	0.5301074706
H	1.1920377781	0.9888646836	-0.8319820557
H	0.6989891734	-0.9328755018	-2.2805252395
H	-1.5881012352	-1.8653538257	-2.3922835597
H	-5.4521827233	0.3043631597	-3.1156290736
H	-6.9921955375	-0.0593786612	-4.9494117219
H	-7.8198066269	-2.2859909561	-5.6289052041
H	-7.0947958253	-4.3069047135	-4.4304225940
H	-3.0146188775	-3.4421793071	0.4308794328
H	-3.2019160378	-5.8562541440	0.5181247939

H	-5.9493712830	-6.0720981675	-2.7567072230
H	-4.6253756211	-7.1803772804	-1.0059983821

CI_{10} (asym)

C	-1.5916076879	-2.9966280136	3.5496420350
C	-1.0477516682	-3.0424893071	2.2839474507
C	-1.8178953201	-2.7556265530	1.1329651702
C	-3.2048921478	-2.3838826233	1.3330542989
C	-3.6891147721	-2.1167494208	2.6440119018
C	-2.8932397387	-2.4994260251	3.7122573340
C	-5.0578862942	-1.7079802343	2.5072215682
C	-5.3926905961	-1.7724663776	1.1234813214
C	-4.2458081880	-2.2260410694	0.3911890501
C	-6.0182028877	-1.3529642000	3.4563667971
C	-7.2964189755	-1.0544395538	3.0487635450
C	-7.6364271081	-1.1288125351	1.6888763492
C	-6.7110067209	-1.4852057892	0.7372484161
C	-3.8988024512	-2.4726749314	-0.9533276575
C	-2.8569543664	-3.3522112372	-1.2260696168
C	-2.3936434827	-3.2006198756	-2.5547695143
C	-3.3398773971	-2.2710463606	-3.2064840793
C	-4.2539069714	-1.8275105235	-2.2273144283
C	-2.1590170078	-4.1200572127	-0.2191271314
C	-0.9418703300	-4.7487608024	-0.7169057402
C	-0.5668537468	-4.6154298012	-2.0132168842
C	-1.2690707833	-3.8259770846	-2.9842648303
C	-5.2146278733	-0.8880402149	-2.5356648306
C	-5.2951219563	-0.4216734015	-3.8479510265
C	-4.4143896908	-0.8773074502	-4.8117688727
C	-3.4169437482	-1.8039417933	-4.4954304407
H	-0.0020794447	-3.2754193894	2.1523146110
H	-1.2799734820	-2.2144139538	0.3677827591
H	-3.2958653014	-2.4380886096	4.7133662387
H	-1.0190794641	-3.2876755458	4.4130734730
H	-5.7570102108	-1.3056531669	4.5028893428
H	-8.0430292004	-0.7685650975	3.7716206161
H	-8.6481666506	-0.9085663928	1.3861281401
H	-7.0100147553	-1.5699695638	-0.2946957914
H	-2.7743135848	-4.7085526583	0.4502047030
H	-0.3760876294	-5.3813088157	-0.0537077877
H	0.3069786272	-5.1577691228	-2.3422212631
H	-0.9015057872	-3.7549082981	-3.9930725081
H	-5.8830327563	-0.5112555696	-1.7813791887
H	-6.0468540146	0.3045561315	-4.1097293270
H	-2.7188888026	-2.1297794564	-5.2495210508
H	-4.4879924985	-0.5027948206	-5.8199247348

REFERENCES

1. Lee, S.; Filatov, M.; Lee, S.; Choi, C. H., Eliminating spin-contamination of spin-flip time dependent density functional theory within linear response formalism by the use of zeroth-order mixed-reference (MR) reduced density matrix. *J. Chem. Phys.* **2018,** *149* (10).
2. Zhu, X.; Thompson, K. C.; Martínez, T. J., Geodesic interpolation for reaction pathways. *J. Chem. Phys.* **2019,** *150* (16).
3. Zahariev, F.; Xu, P.; Westheimer, B. M.; Webb, S.; Galvez Vallejo, J.; Tiwari, A.; Sundriyal, V.; Sosonkina, M.; Shen, J.; Schoendorff, G.; Schlinsog, M.; Sattasathuchana, T.; Ruedenberg, K.; Roskop, L. B.; Rendell, A. P.; Poole, D.; Piecuch, P.; Pham, B. Q.; Mironov, V.; Mato, J.; Leonard, S.; Leang, S. S.; Ivanic, J.; Hayes, J.; Harville, T.; Gururangan, K.; Guidez, E.; Gerasimov, I. S.; Friedl, C.; Ferreras, K. N.; Elliott, G.; Datta, D.; Cruz, D. D. A.; Carrington, L.; Bertoni, C.; Barca, G. M. J.; Alkan, M.; Gordon, M. S., The General Atomic and Molecular Electronic Structure System (GAMESS): Novel Methods on Novel Architectures. *J Chem. Theo. Comp.* **2023,** *19* (20), 7031-7055.
4. Park, W.; Komarov, K.; Lee, S.; Choi, C. H., Mixed-Reference Spin-Flip Time-Dependent Density Functional Theory: Multireference Advantages with the Practicality of Linear Response Theory. *J. Phys. Chem. Lett.* **2023,** *14* (39), 8896-8908.
5. Lee, S.; Park, W.; Choi, C. H., Expanding Horizons in Quantum Chemical Studies: The Versatile Power of MRSF-TDDFT. *Acc. Chem. Res.* **2025,** *58* (2), 208-217.
6. Andersson, M. P.; Uvdal, P., New Scale Factors for Harmonic Vibrational Frequencies Using the B3LYP Density Functional Method with the Triple-ζ Basis Set 6-311+G(d,p). *J. Phys. Chem. A* **2005,** *109* (12), 2937-2941.
7. Jmol: an open-source Java viewer for chemical structures in 3D.
